# Complementary contribution of the medial and lateral human parietal cortex to grasping: a repetitive TMS study

**DOI:** 10.1093/cercor/bhac404

**Published:** 2022-10-15

**Authors:** Rossella Breveglieri, Sara Borgomaneri, Matteo Filippini, Alessia Tessari, Claudio Galletti, Marco Davare, Patrizia Fattori

**Affiliations:** Department of Biomedical and Neuromotor Sciences, University of Bologna, 40126 Bologna, Italy; Center for studies and research in Cognitive Neuroscience, University of Bologna, 47521 Cesena, Italy; IRCCS Santa Lucia Foundation, 00179 Rome, Italy; Department of Biomedical and Neuromotor Sciences, University of Bologna, 40126 Bologna, Italy; Department of Psychology, University of Bologna, 40127 Bologna, Italy; Department of Biomedical and Neuromotor Sciences, University of Bologna, 40126 Bologna, Italy; Faculty of Life Sciences and Medicine, King's College London, SE1 1UL London, United Kingdom; Department of Biomedical and Neuromotor Sciences, University of Bologna, 40126 Bologna, Italy; Alma Mater Research Institute For Human-Centered Artificial Intelligence (Alma Human AI), University of Bologna, Bologna, Italy

**Keywords:** posterior parietal cortex, grasping, transcranial magnetic stimulation, action reprogramming

## Abstract

The dexterous control of our grasping actions relies on the cooperative activation of many brain areas. In the parietal lobe, 2 grasp-related areas collaborate to orchestrate an accurate grasping action: dorsolateral area AIP and dorsomedial area V6A. Single-cell recordings in monkeys and fMRI studies in humans have suggested that both these areas specify grip aperture and wrist orientation, but encode these grasping parameters differently, depending on the context. To elucidate the causal role of phAIP and hV6A, we stimulated these areas, while participants were performing grasping actions (unperturbed grasping). rTMS over phAIP impaired the wrist orientation process, whereas stimulation over hV6A impaired grip aperture encoding. In a small percentage of trials, an unexpected reprogramming of grip aperture or wrist orientation was required (perturbed grasping). In these cases, rTMS over hV6A or over phAIP impaired reprogramming of both grip aperture and wrist orientation. These results represent the first direct demonstration of a different encoding of grasping parameters by 2 grasp-related parietal areas.

## Introduction

The grasping of objects, an exquisite skill of primates, is an action whose complexity is possible thanks to the coordination of different hand movement components, such as wrist orientation and grip aperture (Jeannerod 1988). The capability to perform grasping actions depends on the integrity of networks of areas in the parietal and frontal cortex (Jeannerod et al. 1995; Rizzolatti 1997; Davare et al. 2010; Grafton 2010; Janssen and Scherberger 2015; Galletti and Fattori 2018). Within the monkey parietal lobe, area AIP (anterior intraparietal area, Colby and Duhamel 1991) has a well-known role in grasping (Taira et al. 1990; Sakata et al. 1995; Murata et al. 1996, 2000; Gardner et al. 2007; Baumann et al. 2009; Chen et al. 2009; Schaffelhofer et al. 2015; Schaffelhofer and Scherberger 2016; Lanzilotto et al. 2019, 2020). In the last decades, based on single-cell recordings, area V6A (Galletti et al. 1999) of the dorsomedial visual stream (Galletti et al. 2003; Rizzolatti and Matelli 2003) has been added to the network of areas involved in orchestrating grasping (Galletti et al. 2003; Fattori et al. 2004, 2009, 2010; Breveglieri et al. 2015; Breveglieri et al. 2016; Breveglieri et al. 2018; Filippini et al. 2017). Other techniques, such as neuroimaging (Vesia et al. 2017) and high-resolution 2-deoxyglucose radiography (Evangeliou et al. 2009), have confirmed that V6A is activated by grasping, along with AIP.

A question arises about how similar the functional roles of these parietal areas are in orchestrating grasping. Both V6A and AIP contain cells that are modulated by wrist orientation and grip type (Baumann et al. 2009; Fattori et al. 2009; Breveglieri et al. 2018), suggesting a similar contribution of the 2 areas in grasp encoding. However, some differences emerge in the sensory domain: AIP is richer than V6A in visual cells that encode simple aspects of visual stimuli (Galletti et al. 1999; Gamberini et al. 2011; Romero et al. 2014) and AIP hosts more neurons that are selective for object observation than V6A (Murata et al. 2000; Fattori et al. 2012). V6A also hosts somatosensory cells (Breveglieri et al. 2002) that are not found in AIP (Murata et al. 2000, see Breveglieri et al. 2018 and Maranesi et al. 2014 for a discussion on this topic). These sensory differences can be related to the pattern of connections of the 2 areas, as V6A is not directly connected with the ventral stream (Gamberini et al. 2009; Passarelli et al. 2011), differently from AIP (Borra et al. 2008), and is more strongly connected to somatosensory-related areas. Given these mild differences, it has been suggested that V6A and AIP cooperate in orchestrating how to approach an object to be grasped in the most appropriate way, with V6A likely playing a more relevant role in monitoring the ongoing visuomotor transformation during grasping, and AIP more involved in encoding the geometric features of the object to be grasped (Murata et al. 2000; Maranesi et al. 2014; Schaffelhofer and Scherberger 2016; Fattori et al. 2017; Breveglieri et al. 2018; Galletti and Fattori 2018).

Homologues of areas V6A and AIP have also been described in the human brain. The putative homologue of V6A (hV6A, Pitzalis et al. 2013, 2015) that partially overlaps with area SPOC (Superior Parieto-Occipital Cortex, Cavina-Pratesi et al. 2010; Pitzalis et al. 2015), and the homologue of AIP (putative human AIP, phAIP, Culham et al. 2003, 2006; Frey et al. 2005; Orban 2016) have been studied in several fMRI experiments, but the similarities and differences of the functional features of phAIP and hV6A are still under debate. Most studies did not show large differences in the encoding of grasp-related parameters between phAIP and hV6A (Binkofski et al. 1998; Culham et al. 2003; Begliomini, Caria, et al. 2007; Begliomini, Wall, et al. 2007; Cavina-Pratesi et al. 2007, 2010; Grol et al. 2007; Grafton 2010; Gallivan, McLean, and Culham 2011; Orban 2016). Intriguingly, however, Verhagen and coworkers (Verhagen et al. 2008) found that hV6A is involved in grasping regardless of viewing conditions or object features, whereas phAIP integrates visual information sent by the ventral stream to plan and execute a grasping action. Moreover, fMRI adaptation studies suggest a different activation of lateral and medial parietal regions depending on the grasping parameters (Monaco et al. 2011, 2015).

Transcranial magnetic stimulation (TMS) represents an opportunity to check functional differences between phAIP and hV6A in the encoding of grasping parameters. In a study by Tunik and coworkers (Tunik et al. 2005) where TMS was delivered over phAIP and over a region of the medial parieto-occipital cortex, a disruptive effect was found only after phAIP stimulation. However, the stimulated medial parieto-occipital cortex encompassed more than one cortical area (including both hV6A and the neighboring area hV6) (Tunik et al. 2005), preventing the authors from obtaining conclusive results regarding the causal role of hV6A. In another TMS study, Verhagen (Verhagen et al. 2013) found that regions likely corresponding to phAIP and hV6A specify the same grasping parameters, but the dorsomedial (hV6A) computations depend on the dorsolateral (phAIP) contributions in a sort of parietal hierarchy (Verhagen et al. 2013). Notably, in this study, the TMS pulse was delivered during the *planning* of grasping, and it was found that the stimulation did not alter grasping parameters. It rather altered the alpha-suppression (Verhagen et al. 2013), thus disrupting this index of grasp-related computations.

The specific differences in the causal role of hV6A and phAIP in encoding grip aperture and wrist orientation *during grasping execution* are still largely unknown, and in the present work, we aimed to better understand them by using repetitive TMS (rTMS). We investigated these causal roles during unperturbed grasping movements and during in-flight corrections of grip aperture and wrist orientation in perturbed trials. We found that in unperturbed conditions, rTMS over hV6A interfered with grip aperture, whereas rTMS over phAIP altered wrist orientation. In perturbed grasping, both rTMS over hV6A or over phAIP disrupted grip adjustment and wrist orientation, with higher effects of hV6A on grip aperture and of phAIP on wrist orientation.

## Materials and methods

### Participants

Fifteen healthy volunteers (6 males; age range 19–33 years; mean age 21.93 ± 4.27 years) participated in this study. The number of participants was determined based on a power analysis that indicated that a sample size of 15 participants is an appropriate sample size to achieve statistical power (1−β) of 0.95 (2-tailed α = 0.05; effect size *f* = 0.20, Breveglieri et al. 2021), analysis performed using G*Power software (Faul et al. 2007).

All the participants were right-handed based on the Edinburgh Handedness Inventory (Oldfield 1971), had normal or corrected-to-normal visual acuity in both eyes, and were naïve as to the purposes of the experiment. None of the participants had neurological, psychiatric, or other medical problems or any contraindication to TMS (Rossi et al. 2009; Rossini et al. 2015). Participants provided written informed consent, and the procedures were approved by the Bioethical Committee at the University of Bologna (Prot. 170,133, 21st Nov 2018 and Prot. 237,243, 27 Jul 2020) and were in accordance with the ethical standards of the 2013 Declaration of Helsinki. No discomfort or adverse effects during TMS were reported or noticed.

### Localization of brain sites

To identify the medial (hV6A; Pitzalis et al. 2015) and lateral (phAIP/DIPSA, hereafter called phAIP; Orban 2016) parietal target areas, we used frameless stereotaxic neuronavigation. Before each experimental session, the coil position was identified on the scalp of each participant, who wore a bathing cap with the SofTaxic Navigator system (EMS, Bologna, Italy), using a well-established paradigm (Carducci and Brusco 2012; Tidoni et al. 2013; Paracampo et al. 2017; Valchev et al. 2017; Avenanti et al. 2018). In a first step, skull landmarks (nasion, inion, and 2 preauricular points) and 65 points providing a uniform representation of the scalp were digitized by means of a Polaris Vicra Optical Tracking System (Northern Digital, Inc., Waterloo, ON, Canada). Coordinates in Talairach space were automatically estimated by the SofTaxic Navigator from an MRI-constructed stereotaxic template. This procedure has been proven to ensure a good localization accuracy, showing an error of roughly 5 mm in comparison to methods based on individual MRIs (Carducci and Brusco 2012).

The Talairach coordinates we used were x = −10, y = −78, z = 40 (hV6A) and x = −40, y = −40, z = 40 (phAIP) (Talairach and Tournoux 1988). The coordinates of hV6A are the same as those used in previous TMS studies on this brain region (Ciavarro et al. 2013; Breveglieri et al. 2021) and are similar to those used for studying the SPOC (Vesia et al. 2010, 2013, 2017), a region that likely includes hV6A (Pitzalis et al. 2013; Tosoni et al. 2015; Fattori et al. 2017) and that has been investigated in several imaging studies (Gallivan et al. 2009; Cavina-Pratesi et al. 2010; Gallivan, McLean, and Culham 2011; Rossit et al. 2013; Monaco et al. 2014). The phAIP coordinates were taken from previous neuroimaging studies (Binkofski et al. 1999; Buccino et al. 2001) and are similar to those used in other TMS and neuroimaging studies investigating the role of this same region (Binkofski et al. 1998; Culham et al. 2003; Davare et al. 2007; Cavina-Pratesi et al. 2010; Gallivan, McLean, Valyear, et al. 2011; Vesia et al. 2013). For each participant, the hV6A and phAIP scalp sites were marked on a bathing cap with a pen. Then, the neuronavigation system was used to estimate the projections of the scalp sites on the brain surface. Thus, the position of the center of the coil in our participants was expressed with these mean coordinates ± standard deviation, which corresponded to the left hV6A (mean x = −11.3 ± 2.2, y = −78.8 ± 1.7, z = 41.6 ± 1.9) and to the left phAIP (mean x = −44.9 ± 2.4, y = −43.7 ± 2.2, z = 44.4 ± 3.3). The coil was held by hand. We marked the position of the coil with a pen on the bathing cap and the experimenter continuously checked its position throughout the experiment.

In the monkey, area V6A is buried in the anterior bank of the parieto-occipital sulcus (Galletti et al. 1999), whereas in the human, hV6A lies on the cortical exposed surface of the caudalmost part of the superior parietal lobule, just anterior to the parieto-occipital sulcus (Pitzalis et al. 2013; Pitzalis et al. 2015, for example, see Fig. 5 of Pitzalis et al. 2015). Therefore, TMS over this brain region should be very effective at stimulating hV6A.

The human homologue of monkey area AIP has recently been defined by Orban and coworkers as the cortical region which includes 2 parts called phAIP and DIPSA (Orban 2016). Of these 2 parts, phAIP lies within the intraparietal sulcus and a large amount of DIPSA is located on the cortical exposed surface. With the consideration that TMS stimulation is strongest at the cortical surface (Siebner et al. 2022), we believe to have stimulated the DIPSA in our experiment and, with less stimulation power, the phAIP; in other words, part of the human homologue of monkey area AIP was very probably stimulated. Notably, other TMS studies stimulated the human homologue of monkey area AIP (Tunik et al. 2005; Rice et al. 2006; Davare et al. 2011; Verhagen et al. 2013; Vesia et al. 2013).

### TMS protocol

During each trial, a time-locked single train of 20-Hz rTMS (5 biphasic pulses) was administered using a MagStim Rapid2 stimulator and a 70-mm figure-of-eight coil. The number, frequency, and time-occurrence of the pulses were chosen to interfere with the cortical activity over a time period compatible with the timing of neural activation shown in monkey studies (Murata et al. 2000; Fattori et al. 2010) that demonstrate that the neurons of these areas discharge in the first half of the movement. Sham stimulation was performed by placing the coil tilted at 90° over the vertex, so that no current was induced in the brain (Lisanby et al. 2001). As known from previous experiments (Lisanby et al. 2001; Sandrini et al. 2011), by adopting this procedure, no effective magnetic stimulation reached the brain during the Sham condition, while the participants’ feeling of coil–scalp contact and discharge noise were similar to the real stimulation. We administered sham stimulation over a single site (the vertex) instead of over each of the target areas to provide a single control condition for both hV6A and phAIP sites, i.e. avoiding an increase in the number of trials (Lockwood et al. 2013; Amemiya et al. 2017; Paracampo et al. 2018; Breveglieri et al. 2021).

To set rTMS intensity, the individual resting motor threshold (rMT) was estimated for all participants in a preliminary phase of the experiment using standard procedures (Rossi et al. 2009). Motor-evoked potentials (MEPs) induced by stimulation of the left motor cortex were recorded from the right first dorsal interosseous (FDI) by means of a Biopac MP-36 machine. Electromyography signals were band-pass filtered (30–500 Hz) and digitized at a sampling rate of 5 kHz. Pairs of Ag-AgCl surface electrodes were placed in a belly-tendon montage with a ground electrode on the wrist. The intersection of the coil was placed tangentially to the scalp with the handle pointing backward and laterally at a 45° angle away from the midline. The optimal scalp position for inducing MEPs from the right FDI was first localized, and the rMT was determined from that position. The rMT was defined as the minimal intensity of stimulator output that produced MEPs with an amplitude of at least 50 μV in the FDI with 50% probability (Rossini et al. 2015).

To minimize potential TMS after effects on cortical activity that outlast the period of direct stimulation (Pascual-leone et al. 1994; Wang et al. 1996; Levkovitz et al. 1999; Siebner and Rothwell 2003; Siebner et al. 2009) while keeping the duration of the experiment acceptable, the intertrial period of the task (see below) was randomly set at 5–7 s. Custom software externally triggered the rTMS at movement onset, as explained below (see Behavioral task).

We aimed to stimulate at 120% of rMT, but most of the time, we reduced the intensity because of motor twitches observed when stimulating phAIP, so we actually stimulated on average at 116 ± 5% of rMT.

### Apparatus and behavioral task

**Fig. 1:**
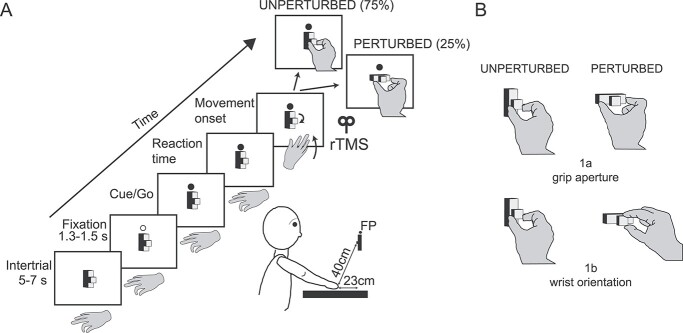
Experimental setup A) left, time sequence of the grasping task. The fixation point (FP, a lit green light-emitting diode, LED, on the panel, here represented in white) stayed on for 1.3 or 1.5 s and then changed its color (from green to red, here represented in black). The participant had to immediately reach and grasp the object with their right hand while maintaining fixation on the FP. The movement onset triggered the rTMS (20 Hz, 5 pulses) and the rotation of the object, from vertical to vertical in the unperturbed trials and from vertical to horizontal in the perturbed trials (for conciseness, we show here only the hand posture observed in Condition 1a). The FP was visible until the participant touched the object. Right, lateral (right) view of the target arrangement in the experimental task. The participants performed grasping movements with their right hand toward the object in a darkened room. B) Drawings of the hand conformation used in the different conditions, in unperturbed and perturbed trials. In perturbed trials, in Condition 1a, an online adjustment of grip aperture was required; in Condition 1b, an online adjustment of wrist orientation was needed.

We tested the influence of rTMS delivered over a medial (likely hV6A) or a lateral (likely phAIP) parietal area on grasping performance by requiring the participants to grasp an object composed of a parallelepiped (1 cm × 1 cm × 5 cm) with a small cube (1 cm × 1 cm × 1 cm) attached at its halfway point, mounted on the shaft of a motor (RS components, UK), and positioned at the level of the subject’s nasion, on a frontal panel (Fig. 1A).

Each trial started, after a variable intertrial period, with a green light-emitting diode (LED), mounted right above the object, coming on. This was the cue for the subject to press a home-button, located close to their chest, with their right hand while fixating the LED. We asked participants to press the home-button with their hand pinched, to obtain a constant initial grip aperture value of around zero before movement onset. Wrist orientation, on the contrary, was not constrained to enable natural grasping strategies. At the start of each trial, the object was oriented vertically. After a fixation period of 1.3–1.5 s (randomly chosen), the LED turned red and this was the “go” signal for the release of the home-button, that in turn triggered 2 devices: the step motor that rotated the object and the TMS stimulator. The participants were instructed to pincer-grasp the object in different ways according to the grasping condition that was run. Condition 1a (Fig. 1B, top) was designed to perturb only the grip aperture. The participant was required to pincer-grasp the little cube with the index finger and thumb opened horizontally. However, if after movement onset the object rotated horizontally (perturbed trial), the subject had to grasp the object’s wider side (the long axis of the object oriented horizontally). Note that in the 2 Conditions, the wrist assumed the same orientation (half-pronated) (see Fig. 1B). In sum, in Condition 1a, the wrist orientation did not change, whereas grip aperture did. Condition 1b (Fig. 1B, bottom), on the contrary, was designed to perturb only the wrist orientation. In this condition, the subject was required to pincer-grasp the little cube. When the object was rotated vertically (unperturbed trials), the wrist was half-pronated, and when the object was rotated horizontally (perturbed trials), the wrist was turned pronated. In both cases, a tiny grip was produced. In sum, in Condition 1b, the grip aperture was always small and constant, whereas wrist orientation was different in unperturbed and perturbed trials.

In unperturbed trials (75% of total trials), the object was rotated 180°, its final orientation remaining vertical. In perturbed trials (25%), the object was rapidly rotated 90° to a horizontal orientation. The type of trial was pseudorandomly selected for each brain site receiving TMS, for each sham stimulation, and for each condition. Although the object was rotated both in 180° and 90° trials, the task goal (size in Condition 1a; orientation in Condition 1b) was perturbed only in the 90° trials. After touching the object, the touch-sensitive tape attached to it signaled to the computer to switch off the fixation point and a new intertrial period started.

The task was composed of 6 blocks of 40 trials each (30 unperturbed and 10 perturbed per block) for a total of 240 trials performed over the same experimental session. Each session lasted ~2 h. Two blocks involved active stimulation of the medial parietal site (hV6A), 2 active stimulations of the lateral parietal site (phAIP), and the remaining 2 sham stimulations. We randomized blocks of each Stimulation site and of each task condition (hV6A, phAIP, Sham, 1a, 1b). The grasping movements were performed in a darkened room. The head of the participant was supported by a head-chin rest to reduce head movements. To run the task and for data analysis, we used Matlab (Mathworks, USA). No participant reported the presence of scotoma or phosphenes in the visual field during the sessions.

### Data acquisition, analysis, and statistics

The kinematics of grasping movements was recorded using a motion tracking system (VICON motion capture system, 6 Vero 2.2 cameras, 2.2MP, 2048 × 1088 pixel resolution) by sampling the position of 4 markers at a frequency of 100 Hz; markers consisted in balls mounted on a band attached to the skin of the wrist (on the ulnar styloid process and on the radial styloid process) and of the tips of the right index finger and of the thumb.

Movement onset/offset were determined as the moment when the markers’ velocities (specifically the earliest chosen from the index or thumb) exceeded/fell and remained below 30 mm/s. Movement time was obtained by subtracting the movement onset from the respective movement offset. Participants were asked to move their hand in a ballistic way (without pauses or interruptions), at a fast but comfortable speed, and as accurately as possible.

Eye position was recorded using a camera-based eye-tracker (Pupil Lab, Pupil Labs Gmbh) recording real-time gaze position at 200 Hz.

As noticed above, Condition 1a was specifically set up to manipulate grip aperture, which was defined as the 3D distance between the index and thumb markers. Time to maximum grip aperture (tMGA) was defined as the time during the movement where the maximum value of grip aperture (MGA) was reached. Condition 1b was specifically set up to manipulate wrist orientation, which was defined as the angle formed by the plane connecting the markers of the wrist and the index tip and the horizontal plane.

To evaluate whether the stimulation had any effects on MGA, tMGA, and movement time, and whether these effects were specific for trial type, we used 3 separate 2-way repeated-measures ANOVAs [factors: Stimulation site (Sham, phAIP, hV6A), Trial Type (unperturbed, perturbed)] in line with other reports (e.g. Tunik et al. 2005; Breveglieri et al. 2021).

To investigate the time-course of the effect of stimulation on grip aperture or on wrist angle, we divided the grip aperture data and the wrist angle values into 10% time bins relative to the tMGA and thus performed 2 separate 3-way repeated measures ANOVA with the following factors: Stimulation site (phAIP, Sham, hV6A), Trial type (perturbed, unperturbed), and time Bin (13 levels, 10th bin ending at tMGA).

All post-hoc comparisons were carried out with the Duncan test to consider corrections for multiple comparisons. Effect size indices for main effects and interactions were computed using partial eta squared (partial η^2^), whereas repeated measures Cohen’s *d* was computed for post-hoc comparisons by dividing the difference of the mean values of the 2 groups by the standard deviation of differences of the group elements (Cohen 1977; Wolf 1986).

Since our aim was to assess the critical role of the medial and lateral parietal areas hV6A and phAIP in grasping movements, in the text, we report only the main effects or the interactions of/by the Stimulation site.

## Results

In our experiment, participants performed grasping movements of an object whose orientation required them to adjust the grip aperture (Condition 1a) or the wrist orientation (Condition 1b) online while stimulating, with rTMS, 2 different sites of the parietal cortex known to be grasp-related. We hereafter present the effects of the stimulation of either the medial (hV6A) or the lateral (phAIP) parietal site on different grasp-related parameters in each Condition.

### Effects on grip aperture

**Fig. 2:**
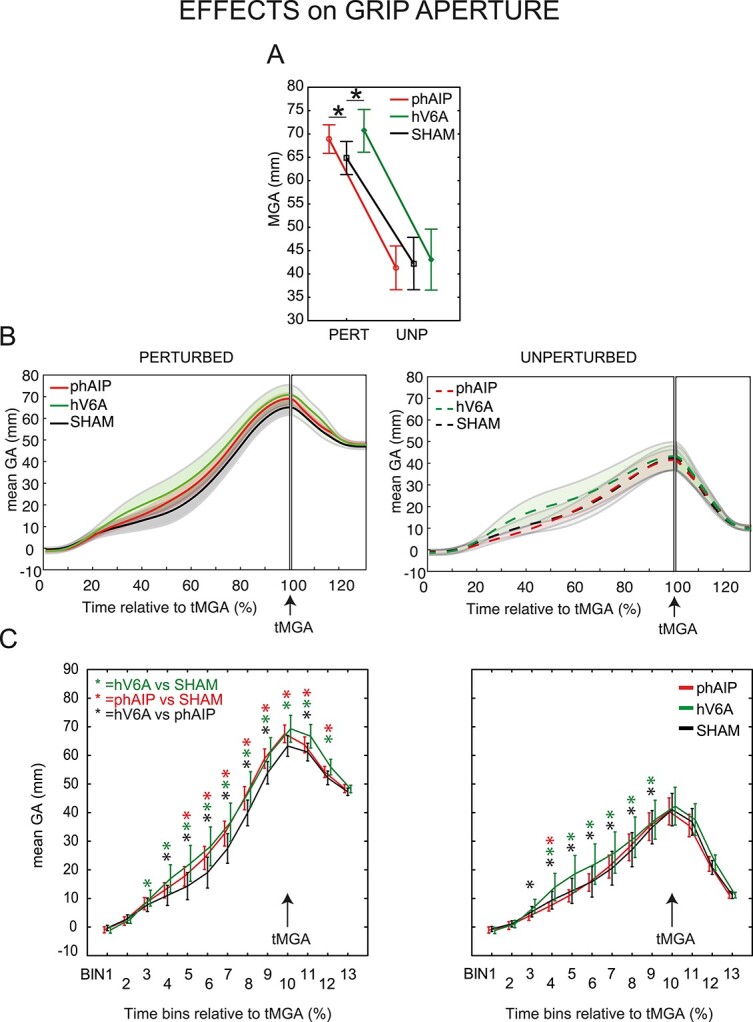
Effects of rTMS on grip aperture (Condition 1a). A) MGA values for the different stimulation sites (phAIP, red; hV6A, green; sham, black) in the different trial types (PERT, perturbed; UNP, unperturbed). A significant increase in the MGA is evident after phAIP or hV6A stimulation compared with sham stimulation. Asterisks indicate significant posthoc comparisons (see methods for specifications). B) Grip aperture averaged over participants for each of the 6 conditions from movement onset to the tMGA. Participants started the movement with their hand pinched, and grip apertures were wider in perturbed trials, as expected. Dashed curves (right) represent unperturbed trials, continuous lines (left) represent perturbed trials. Same color specifications as in a). C) Mean grip aperture for each of the 3 stimulation sites (phAIP, red; hV6A, green; sham, black) in each of the trial types (perturbed, left; unperturbed, right). The data have been grouped into 10% time bins in terms of tMGA, demonstrating significant differences in grip aperture between stimulation sites and trials over time. Colored asterisks represent significant posthoc comparisons (perturbed trials: phAIP vs. sham *P* < 0.001 in bin 5, *P* < 0.00001 in bins 6–10, *P* < 0.05 in bins 11–12; phAIP vs. hV6A, *P* < 0.0001 in bins 4–7, *P* < 0.05 in bins 8, 9, 11; hV6A vs. sham, *P* < 0.05 in bin 3, *P* < 0.001 in bin 12, *P* < 0.00001 in bins 4–11; unperturbed trials phAIP vs. sham *P* < 0.05 in bin 4; phAIP vs. hV6A, *P* < 0.001 in bins 3 and 8, *P* < 0.05 in bins 9, *P* < 0.00001 in bins 4–7; hV6A vs. sham, *P* < 0.05 in bin 9, *P* < 0.00001 in bins 4–8). Vertical bars represent SE. A deviation from Sham is evident and statistically significant both in unperturbed and perturbed trials after hV6A stimulation, whereas after phAIP stimulation, a deviation is evident only in perturbed trials.

We investigated whether MGA and tMGA were affected by the Stimulation Site and/or by Trial Type. We observed a significant interaction effect of Stimulation Site by Trial Type on MGA (Fig. 2A and Tables 1 and S1, *F*_2,28_ = 6.72; *P* < 0.01; partial η^2^ = 0.32, individual participants’ data are shown in Fig. S1A). This effect was driven by the increase in the MGA following the stimulation of each of the parietal sites only in perturbed trials of Condition 1a (phAIP vs. hV6A *P* = 0.12, Cohen’s *d* = 0.21) compared with Sham (Sham vs. phAIP *P* = 0.001, Cohen’s *d* = 0.77, Sham vs. hV6A *P* < 0.001, Cohen’s *d* = 0.80). When no online correction was required (unperturbed trials), no significant post-hoc comparisons were found (Fig. 2A, all *P* > 0.14, all Cohen’s *d* < −0.18). This suggests that, during perturbed grasping, rTMS over either the lateral or the medial parietal areas during grasping execution interfered with the online control of grip aperture. No significant effect of the Stimulation Site was found on tMGA (Tables 2 and S2, all *F* < 1.57; all *P* > 0.22; all partial η^2^ < 0.10).

**Table 1:** Mean MGA of Condition 1a across trials and variability (standard errors, SE) for each stimulation site and trial type.

Stimulation site	Trial type	MGA (mm)	SE (ms)
SHAM	unperturbed	42.41	5.44
phAIP	unperturbed	41.53	4.52
hV6A	unperturbed	43.26	6.31
SHAM	perturbed	65.05	3.55
phAIP	perturbed	69.11	3.06
hV6A	perturbed	70.87	4.59

**Table 2:** Mean tMGA of Condition 1a across trials and variability (SE) for each stimulation site and trial type.

Stimulation site	Trial type	tMGA (%MT)	SE (ms)
SHAM	unperturbed	61.01	2.62
phAIP	unperturbed	58.33	3.01
hV6A	unperturbed	59.85	2.39
SHAM	perturbed	68.36	1.55
phAIP	perturbed	65.95	1.86
hV6A	perturbed	67.60	1.69

As commonly seen in grasping, grip aperture changes throughout the entire grasping action (Fig. 2B). In perturbed trials, we observed an increase in grip aperture before the tMGA following phAIP and hV6A stimulations, but after hV6A stimulation, the effect was more pronounced (see the green curve on the left of Fig. 2B). This predominance of hV6A effect is reduced, but still present, toward tMGA, and continues after the tMGA up until when the grip closes around the object. The effect disappears just before the contact with the object. In unperturbed trials, an increase in grip aperture before the tMGA was only observed following hV6A stimulation, while phAIP stimulation, compared with the Sham condition, seems not to affect grip aperture (see the dashed green curve on the right of Fig. 2B). The hV6A effect disappears while approaching the tMGA and it is not present when the grip closes around the object.

To quantify these differences over time, we divided the grip aperture data into 13 bins, each one comprising 10% of movement time required to reach the MGA (the 10th bin ending at tMGA, Fig. 2C, individual participants’ data in Fig. S1B), and we performed a repeated measures 3-way ANOVA. We found a significant Stimulation Site by Trial Type by Bin interaction (Table S3, *F*_(24,336)_ = 1.77; *P* = 0.02; partial η^2^ = 0.11, Fig. 2C, see Fig. S3A for Cohen’s *d*), which suggests a causal, time-dependent involvement of hV6A and phAIP in perturbed trials and the involvement of hV6A in unperturbed trials (all the posthoc comparisons are listed in the figure legend). At the beginning of the perturbed trials (from 30 to 50% of the time relative to tMGA), there was a significant increase in grip aperture following the stimulation over hV6A compared with Sham and phAIP and also following the stimulation over phAIP vs. Sham; from 50 to 90% of the time, a differential effect of hV6A and phAIP stimulation was observed, which suggests a stronger and earlier contribution of hV6A, compared with phAIP, to finger configuration during grasping. Also, the contributions of hV6A and of phAIP during grip closure after the tMGA were statistically confirmed, with a major contribution of hV6A at the beginning of the closure (see bin 11 of Fig. 2C, left). In unperturbed trials (Fig. 2C, right), a significant major impairment of grip aperture emerges from the stimulation of hV6A (from 30 to 90% of the time compared with tMGA) but not of phAIP, while no effects of either stimulation site were found after tMGA.

No significant main effects of the Stimulation Site were found, nor interactions of Stimulation Site by Trial Type on movement time (Table S4, all *F* < 0.92; all *P* > 0.40; all partial η^2^ < 0.06; see Table 3 for mean values), suggesting that the increase in grip aperture caused by hV6A and phAIP stimulation was not associated with changes in action timing.

**Fig. 3:**
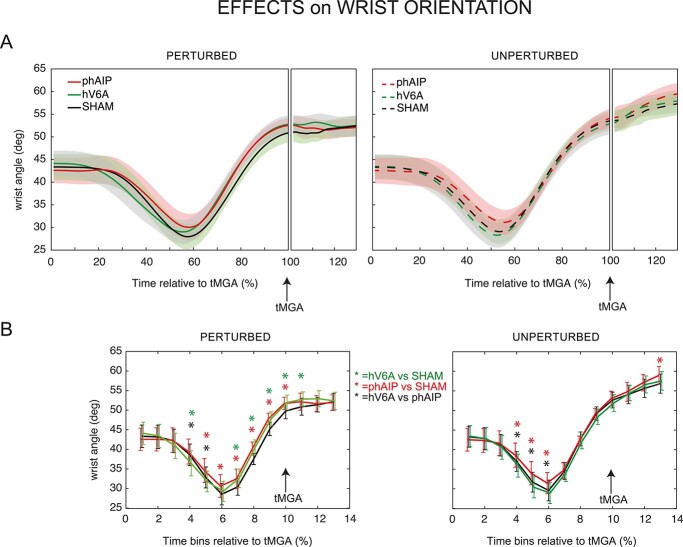
Effects of rTMS on wrist orientation (condition 1b). A) Wrist orientation (expressed as wrist angle) in the different stimulation sites and different trial types averaged over participants from movement onset to movement end. Same line specifications as in Fig. 2. B) Mean wrist angle for each of the 3 stimulation sites in each of the trial types. The data have been grouped into 10% time bins in terms of tMGA. Other conventions as in Fig. 2B–C.

### Effects on wrist orientation

To check whether these two parietal regions are also involved in the adjustments of wrist orientation, in different, but randomized, blocks of trials, we instructed the participants to grasp only the small cube on the object, using different wrist orientations depending on the orientation of the object. As a result, we observed that the participants unexpectedly corrected wrist orientation while maintaining the same grip aperture (Condition 1b).

By means of a repeated measures 2-way ANOVA with Stimulation Site and Trial Type as factors, we did not find any significant main effect of Stimulation Site or interaction of Stimulation Site by Trial type on movement time (Table S5, all *F* < 1.30; all *P* > 0.28; all partial η^2^ < 0.08; see Table 3 for mean values), and this suggests that the movement time of this Condition was not influenced by rTMS either in unperturbed or in perturbed trials. Therefore, neither phAIP nor hV6A are generally involved in the timing of an action requiring wrist orientation adjustments to grasp an object.

To further investigate this topic along the duration of the movement, consistently with the analysis of grip aperture data, we compared the profiles of wrist angle over time in the different stimulation sites and trial types using a 3-way repeated measures ANOVA with the factors Stimulation Site, Trial Type, and time Bin. We found a significant interaction of Stimulation Site by Trial Type by time Bin, (Table S6, *F*_(24,336)_ = 1.59; *P* = 0.04; partial η^2^ = 0.10, see Fig. S3b for Cohen’s *d and individual participants’ data in Fig. S2*). The effects consisted in a reduction of the wrist angle, indicative of a lesser wrist rotation, observed in the intermediate parts of the movement of perturbed trials after hV6A or phAIP stimulation (hV6A vs. Sham, bin 4 and bins 7–11, all *P* < 0.03, phAIP vs. Sham, bins 5–10, all *P* < 0.04, hV6A vs. phAIP, bins 4–5, all *P* < 0.001, all other bins *P* > 0.09, Fig. 3A and B, left), whereas at the beginning and end of the movement, no such changes were observed (all *P* > 0.05). These data suggest that both hV6A and phAIP are involved in wrist orientation reprogramming. In unperturbed trials, significant changes in wrist angle were found only after phAIP stimulation (hV6A vs. Sham, all *P* > 0.05, phAIP vs. Sham, bins 4–6 and bin 13, all *P* < 0.04, hV6A vs. phAIP, bins 4–6, all *P* < 0.001, Fig. 3A and B, right).

To summarize, the present data suggest that the stimulation of phAIP and hV6A produced significant effects in perturbed trials, mainly in the intermediate part of the movement during which wrist adjustments are required to approach the object or to correct online the wrist orientation following an unexpected perturbation of object orientation. Differently, in unperturbed trials, only the stimulation of phAIP produced consistent effects in the adjustments of wrist orientation.

## Discussion

In this study, we looked for the involvement of phAIP and hV6A in the execution of the grasping action. We used rTMS over these 2 parietal areas during movement execution and found different effects on kinematic measures associated with hand preshaping in a delayed grasping task in which the transport component was kept constant. Specifically, our findings suggest that hV6A and phAIP are involved in grip aperture and in wrist rotation when grasp reprogramming is required, whereas, when grasping is unperturbed, hV6A is involved in grip aperture and phAIP in wrist orientation.

**Table 3:** Mean movement times across trials and variability (SE) for each stimulation site, Condition, and trial type.

Stimulation site	Condition	Trial type	Movement time (ms)	SE (ms)
SHAM	1a	unperturbed	1017.37	41.59
phAIP	1a	unperturbed	1017.66	41.03
hV6A	1a	unperturbed	1028.67	51.02
SHAM	1a	perturbed	1085.74	46.29
phAIP	1a	perturbed	1109.03	46.89
hV6A	1a	perturbed	1115.63	51.09
SHAM	1b	unperturbed	1080.47	56.56
phAIP	1b	unperturbed	1112.85	60.32
hV6A	1b	unperturbed	1030.63	43.89
SHAM	1b	perturbed	1045.91	67.57
phAIP	1b	perturbed	1061.68	51.68
hV6A	1b	perturbed	1023.46	47.61

We actively stimulated 2 cortical areas in addition to sham stimulation, and this enabled us to rule out the possibility of nonspecific effects of the stimulation, for example that the increase in grip aperture was a functional response (e.g. wider safety margin) rather than an effect of the disruption per se. The differential effects of rTMS in hV6A and phAIP in the 2 trial types suggest that the changes in grip aperture represent an impairment induced by TMS specifically in a cortical area and are informative as to its causal role in grasping execution.

### The causal role of phAIP and of hV6A in grasping execution

The dorsolateral parietal grasping area has been extensively studied by neurophysiological experiments in the monkey brain (Taira et al. 1990; Sakata et al. 1995; Murata et al. 1996, 2000; Gardner et al. 2007; Baumann et al. 2009; Chen et al. 2009; Schaffelhofer et al. 2015; Schaffelhofer and Scherberger 2016; Lanzilotto et al. 2019, 2020) and in the human brain by the use of fMRI (Grafton et al. 1996; Binkofski et al. 1998; Culham et al. 2003; Frey et al. 2005; Begliomini, Caria, et al. 2007; Begliomini, Wall, et al. 2007; Grol et al. 2007; Cavina-Pratesi et al. 2007, 2010; Verhagen et al. 2008; Hinkley et al. 2009; Grafton 2010; Gallivan, McLean, and Culham 2011; Orban 2016) and TMS (Tunik et al. 2005; Rice et al. 2006; Davare et al. 2011; Verhagen et al. 2013; Vesia et al. 2013). The grip aperture and wrist orientation impairments shown here are in line with these studies and, furthermore, suggest that phAIP is strongly involved in the monitoring of wrist orientation, independently from the task context. Given the complexity of the wrist joint (Linscheid 1986) and its influence on the determination of optimal grip aperture and affordances (Hazelton et al. 1975; Johnston et al. 2010), the major involvement of phAIP in the wrist orientation process fits well with the model proposed by Bonaiuto and Arbib (Bonaiuto and Arbib 2015) in which phAIP plays a pivotal role in affordance extraction based on the information regarding object features sent by hV6A. Again in line with this model is the delayed contribution shown here by phAIP (compared with that of hV6A) during grasping (see, for example Fig. 2C left, in which the impairments after stimulation of phAIP are delayed compared with those of hV6A). The present results are also in line with those of Tunik (Tunik et al. 2005) showing that phAIP is involved in grip aperture adjustments only in perturbed conditions.

Similarly to AIP, several neurophysiological studies have shown the existence of grasp-related neurons in the monkey V6A (Fattori et al. 2009, 2010; Breveglieri et al. 2016, 2018) and fMRI studies of grasp-related activation in a region of the human brain that likely includes the human homologue of the monkey V6A (Verhagen et al. 2008; Gallivan, McLean, Valyear, et al. 2011; Monaco et al. 2011, 2015; Gutteling et al. 2015; Styrkowiec et al. 2019; Sulpizio et al. 2020). Both results are in line with the results presented here. Moreover, V6A lesion in the monkey produced misreaching, but also misgrasping; abnormal grip aperture and wrist orientation were seen when the animal attempted to grasp pieces of food (Battaglini et al. 2002). This impairment was also evident in patients with lesions that likely included hV6A (Perenin and Vighetto 1988; Karnath and Perenin 2005). Thus, current results strongly support the functional homology between monkey and human medial parietal cortex.

Differently from phAIP, only a few stimulation studies on hV6A during grasping have been performed. In Verhagen’s TMS-EEG study (Verhagen et al. 2013), the stimulation of hV6A produced an alteration in alpha suppression during the planning of grasping of differently slanted objects but did not alter any grasping parameters compared with the control condition (TMS on the Vertex) (Verhagen et al. 2013). In Vesia’s studies (Vesia et al. 2013; Vesia et al. 2017), paired pulse TMS showed that SPOC influences primary motor cortex during grasp planning. None of these studies demonstrated alterations in grasping execution following TMS over hV6A. Thus, the current results are the first demonstrating the causal role of hV6A in grasping execution using a virtual lesion protocol.

We show here that hV6A is primarily involved in grip aperture adjustment, which is directly related to an intrinsic property of the object as its dimension. It is also known that hV6A processes the spatial location of objects (see Galletti et al. 2003 and Fattori et al. 2017 for reviews), an extrinsic object property. Thus, we suggest that during movement execution hV6A sends intrinsic and extrinsic object properties to phAIP (thanks to direct interconnections demonstrated in the monkey and in the human brain, Borra et al. 2008; Tosoni et al. 2015) an area which, in turn, analyzes object properties to extract affordances, in line with the neural model of Bonaiuto and Arbib (Bonaiuto and Arbib 2015). This suggestion, however, is not in line with the supposed view of phAIP making an earlier contribution to grasping, compared with hV6A, shown in the study by Verhagen and coworkers (Verhagen et al. 2013). But since the TMS was delivered during movement planning in Verhagen’s study, whereas in our study, it was delivered during movement execution, we suggest that during planning the phAIP sends input to hV6A, whereas during execution the hV6A sends input to the phAIP. The presence of direct bidirectional connections between AIP and V6A, as demonstrated in the macaque brain (Borra et al. 2008; Gamberini et al. 2009), provides this speculation with anatomical support.

The part of hV6A we stimulated here likely corresponds to the dorsal part of hV6A (hV6Ad), which is the part of hV6A that is active during real and imagined grasping (Sulpizio et al. 2020) and the impairments observed here after TMS over hV6A are in line with recent decoding studies demonstrating that V6A neural signals can be successfully used to decode grasp-related information (Filippini et al. 2017; Nelissen et al. 2018). A potential limit of all TMS studies is that the magnetic field may spread to the areas located close to the stimulated one. In our case, for instance, it is plausible that the stimulation of phAIP might have spread over Brodmann’s area 2 and that the stimulation over hV6A spread over early visual areas. If this were the case, the TMS-induced noise in the somatosensory system could have disturbed the reprogramming of finger movements after phAIP stimulation, and/or the TMS-induced noise in the visual cortex could have caused visual disturbance after hV6A stimulation. We think we can exclude this possibility because, if this had been the case, the effect of spurious stimulation should have affected the grip aperture and/or the wrist orientation in both perturbed and unperturbed trials. This, however, was not the case.

### Dorsomedial and dorsolateral contributions to the specification of grasping parameters

The 2-channels hypothesis postulated by Jeannerod (Jeannerod 1988) suggests that the 2 components of the reach-to-grasp action (transport and grip components) are processed by 2 different substreams within the dorsal visual stream: the dorsomedial substream that processes the transport component and the dorsolateral one that processes the grip component. Despite support for this hypothesis derived from neuroimaging (Cavina-Pratesi et al. 2010), neuropsychology (Binkofski et al. 1998), and single-cell recording studies (Jeannerod et al. 1995), a new radical idea has been established in the last decades according to which both substreams encode the transport and the grip components of reach-to grasp actions (Battaglini et al. 2002; Fattori et al. 2004, 2010), confirmed by additional studies (Monaco et al. 2011, 2015; Verhagen et al. 2013; Breveglieri et al. 2016, 2018). Specifically, single-cell recordings in the monkey showed that both AIP and V6A are grasp-related (Galletti et al. 2003; Fattori et al. 2004, 2010, 2012; Maranesi et al. 2014; Janssen and Scherberger 2015; Breveglieri et al. 2016, 2018), with both AIP and V6A similarly linked to the geometric, intrinsic features of the object to be grasped, but with V6A changing its encoding scheme from object observation to grasping execution, when the link to the geometric features slightly disappears in favor of grip encoding (Murata et al. 2000; Fattori et al. 2012). The current results support the view that both dorsomedial and dorsolateral circuits in monkeys and humans encode the same functional parameters of grasping. We also demonstrate that the dorsomedial (hV6A) and the dorsolateral (phAIP) parietal cortex participate differently in the processing of grip aperture and wrist orientation during grasp execution either when the motor plan remains unaltered or when it needs to be suddenly changed.

The impairments in grip aperture only during perturbed trials after stimulation of phAIP were also found in the TMS study of Tunik and coworkers (Tunik et al. 2005), whereas in that of Rice and coworkers (Rice et al. 2006), phAIP stimulation produced impairments in grip aperture during both perturbed and unperturbed trials. The discrepancy could be caused by the different task design. In Rice’s study, grasp-related visual information was not available and the percentage of perturbed trials was 50%, whereas in Tunik’s and in our study, visual information about the arm was available and the incidence was much lower (25%). Since it has been demonstrated that only a percentage of around 20% makes any perturbation truly unexpected (Posner 1980; Paulignan et al. 1991; Rumiati and Humphreys 1998; Pisella et al. 2000; Tessari and Rumiati 2004; Jacquet et al. 2012; Song et al. 2014), the possibility exists that the perturbation in Rice’s study was somehow “expected” by participants, and this might explain the observed differences in results.

These results agree with the findings of 2 neuroimaging studies that employed the functional magnetic resonance imaging adaptation paradigm (Monaco et al. 2011, 2015). First, a reduced fMRI activation (adaptation) was found in the human aIPS (likely corresponding with phAIP) and in SPOC (likely corresponding with hV6A) to object size (Monaco et al. 2015), demonstrating that both areas participate in the processing of grip aperture. Our results regarding wrist orientation encoding are similarly in line with functional neuroimaging, since it has been reported that the anterior SPOC (partially overlapping with hV6A) signal decreases after repetitive grasping with the same wrist orientation (Monaco et al. 2011), which indicates that SPOC is involved in wrist orientation processing.

Overall, both the current results and those from single-cell recordings and fMRI experiments go against the traditional notion concerning the existence of a medial-to-lateral segregation of reaching versus grasping encoding.

### Possible effect of task conditions

The present results might also be interpreted in the light of the different grasping contexts of the task conditions. In Condition 1a, participants grasped different objects in unperturbed (small cube) vs. perturbed (long parallelepiped) trials, i.e. the goal of the action and the intrinsic properties of the target object changed between trials. The involvement of hV6A in both trial types could thus be informative of a causal role of this area in grip aperture encoding, irrespectively of the goal of the action or of the intrinsic properties of the object. In the phAIP, on the contrary, the grip aperture encoding seems to be specifically linked to the goal of the action or to the intrinsic grasping properties of the object, in line with other studies (Tunik et al. 2005; Romero et al. 2014). In fact, the phAIP seems to act in a context where a grip aperture adjustment is accompanied by a sudden change in the goal of the action. In Condition 1b, the goal of the action is invariant, whereas the affordance changes. Since in this condition both phAIP and hV6A are involved in perturbed trials, when a reprogramming of affordance is requested, it seems that the encoding of wrist orientation in both phAIP and hV6A is linked to the affordance (as also shown for the macaque V6A, Breveglieri et al. 2015). On the other hand, the encoding of wrist orientation in the phAIP could also be influenced by intrinsic object properties, because phAIP is also involved in unperturbed trials. Therefore, these data suggest that the lateral and medial parietal grasping areas could encode the kinematic parameters differently, depending on the context.

## Conclusions

The present experiment was designed to map the specific grasp-related role of hV6A and phAIP and to contribute to the debate regarding the functional role of the dorsomedial and the dorsolateral grasping areas. We found that the 2 regions collaborate in providing information to the frontal lobe in terms of grip aperture and wrist orientation in a context-dependent manner. In fact, hV6A and phAIP are both critically involved in the integration of wrist orientation and grip aperture when orchestrating a grasping action for which there is an explicit need to update the movement. When there is no need of motor reprogramming, hV6A processes information mostly related to grip aperture, whereas phAIP processes information regarding mostly wrist orientation. Our findings support previous studies suggesting that both parietal regions are involved in grasping, by specifying the same grasping parameters in a different way according to the context. These results could be useful for establishing rehabilitation protocols in the case of brain damage as well as in the use of phAIP and hV6A neural signals to specify the appropriate grasping parameters in the generation of accurate movements within a brain–computer interface.

## Funding

European Union’s Horizon 2020 research and innovation programme under grant agreement No 951910. This article reflects only the author’s view, and the Agency is not responsible for any use that may be made of the information it contains.

## Conflict of interest statement

The authors declare no conflicts of interests.

## Supplementary Material

Supplementary_material_bhac404
