# Supplementary material for: Complementary contribution of the medial and lateral human parietal cortex to grasping: a repetitive TMS study
Source: Cereb Cortex. 2022 Oct 15;33(9):5122–34. doi: 10.1093/cercor/bhac404 (PMC10152058; doi:10.1093/cercor/bhac404)
Supplement: Supplementary_material_bhac404 [file supplementary_material_bhac404.docx]

**Supplementary material**

**Complementary contribution of the medial and lateral human parietal cortex to grasping: a repetitive TMS study.**

**Breveglieri Rossella^1^*, Borgomaneri Sara^2.3^, Filippini Matteo^1^, Tessari Alessia^4^, Galletti Claudio^1^, Davare Marco^5^, Fattori Patrizia^1.6^.**

1. Department of Biomedical and Neuromotor Sciences. University of Bologna, 40126 Bologna, Italy

2. Center for studies and research in Cognitive Neuroscience, University of Bologna, 47521 Cesena, Italy.

3. IRCCS Santa Lucia Foundation, 00179 Rome, Italy.

4. Department of Psychology, University of Bologna, 40127 Bologna, Italy.

5. Brunel University, London, UK.

6. Alma Mater Research Institute For Human-Centered Artificial Intelligence (Alma Human AI), University of Bologna.

**Fig. S1**

A) Individual participants’ differences in MGA between phAIP-SHAM and hV6A-SHAM (gray squares) and mean values across participants (black filled squares). B) Individual participants’ grip aperture differences between phAIP-SHAM (top) and hV6A-SHAM (bottom) (gray squares) and mean values (black filled squares) for each time bin relative to tMGA in perturbed and unperturbed trials.

**Fig. S2**

Individual participants’ wrist orientation (expressed as wrist angle) after phAIP stimulation (top) hV6A (center), and Sham stimulation (bottom). Other conventions as in Fig. S2B.

**Fig. S3**

Cohen’s d for each significant posthoc comparison of data shown in Figure 2C (A) and 3B (B) for each time bin relative to tMGA.

Table S1

Statistical effects on MGA (Condition 1a). In bold significant differences are shown.

|  | F | p | Partial η2 |
| --- | --- | --- | --- |
| Stimulation Site | 1.90 | 0.17 | 0.12 |
| Trial Type | **72.36** | **0.00** | **0.84** |
| Stimulation Site by Trial Type | **6.72** | **0.00** | **0.32** |

Table S2

Statistical effects on tMGA (Condition 1a). Other conventions as in Table S1.

|  | F | p | Partial η2 |
| --- | --- | --- | --- |
| Stimulation Site | 1.58 | 0.22 | 0.10 |
| Trial Type | **34.32** | **0.00** | **0.71** |
| Stimulation Site by Trial Type | 0.04 | 0.96 | 0.00 |

Table S3

Statistical effects on grip aperture (Condition 1a). Other conventions as in Table S1.

|  | F | p | Partial η2 |
| --- | --- | --- | --- |
| Stimulation Site | 2.43 | 0.11 | 0.15 |
| Trial Type | **178.15** | **0.00** | **0.93** |
| Bin | **68.21** | **0.00** | **0.83** |
| Stimulation Site by Trial Type | **5.46** | **0.00** | **0.28** |
| Stimulation Site by Bin | **2.23** | **0.00** | **0.14** |
| Trial Type by Bin | **81.24** | **0.00** | **0.85** |
| Stimulation Site by Trial Type by Bin | **1.77** | **0.02** | **0.11** |

Table S4

Statistical effects on movement time (Condition 1a). Other conventions as in Table S1.

|  | F | p | Partial η2 |
| --- | --- | --- | --- |
| Stimulation Site | 0.36 | 0.70 | 0.02 |
| Trial Type | **20.87** | **0.00** | **0.60** |
| Stimulation Site by Trial Type | 0.93 | 0.41 | 0.06 |

Table S5

Statistical effects on movement time (Condition 1b). Other conventions as in Table S1.

|  | F | p | Partial η2 |
| --- | --- | --- | --- |
| Stimulation Site | 1.30 | 0.29 | 0.085 |
| Trial Type | 3.66 | 0.08 | 0.21 |
| Stimulation Site by Trial Type | 0.91 | 0.41 | 0.06 |

Table S6

Statistical effects on wrist angle (Condition 1b). Other conventions as in Table S1.

|  | F | p | Partial η2 |
| --- | --- | --- | --- |
| Stimulation Site | 0.38 | 0.69 | 0.03 |
| Trial Type | 2.87 | 0.11 | 0.17 |
| Bin | **24.48** | **0.00** | **0.64** |
| Stimulation Site by Trial Type | 2.58 | 0.09 | 0.16 |
| Stimulation Site by Bin | 0.73 | 0.83 | 0.05 |
| Trial Type by Bin | **3.52** | **0.00** | **0.20** |
| Stimulation Site by Trial Type by Bin | **1.59** | **0.04** | **0.10** |
